# Black Pigmentation on the Tongue Induced by Long-Term Use of Tetracycline Antibiotics in a Colorectal Cancer Patient With Epidermal Growth Factor Receptor (EGFR) Inhibitor-Associated Skin Lesions: A Case Report and Literature Review

**DOI:** 10.7759/cureus.79261

**Published:** 2025-02-18

**Authors:** Takahito Inagaki, Takenori Ichimura, Daisuke Ichikura, Kunihiko Wakamura, Shuichi Nawata

**Affiliations:** 1 Department of Pharmacy, Showa University Northern Yokohama Hospital, Yokohama, JPN; 2 Division of Hospital Pharmaceutics, Showa University School of Pharmacy, Tokyo, JPN; 3 Department of Pharmacy, Showa University Fujigaoka Hospital, Yokohama, JPN; 4 Digestive Disease Center, Showa University Northern Yokohama Hospital, Yokohama, JPN

**Keywords:** black pigmentation on the tongue, colorectal cancer, egfr inhibitor, panitumumab, skin lesions, tetracycline antibiotics

## Abstract

Skin lesions are prevalent in patients with metastatic colorectal cancer (mCRC) receiving epidermal growth factor receptor (EGFR) inhibitors, such as panitumumab and cetuximab, often necessitating long-term management. Despite adequate treatment involving topical adrenocorticosteroid medications, severe skin lesions may result in prolonged or discontinued EGFR inhibitor administration. Consequently, tetracycline antibiotics, with their antibacterial and anti-inflammatory effects, are recommended for EGFR inhibitor-induced acne dermatitis. We present a 68-year-old male patient with mCRC who underwent chemotherapy with panitumumab, folinic acid, 5-fluorouracil, and irinotecan but subsequently exhibited black pigmentation on the tongue due to six months of prolonged minocycline administration for treating panitumumab-induced skin lesions. Suspecting minocycline-related adverse effects based on the description and a score of 7 on the Naranjo scale, minocycline was discontinued while other medications were maintained. The color of the dorsal tongue gradually normalized within two weeks after discontinuing minocycline. Presently, chemotherapy is continued, whereas panitumumab is repeatedly started and stopped according to the severity of acneiform efflorescence and paronychia. Panitumumab was continued without dose reduction or discontinuation owing to the suppression of skin lesions by minocycline; however, the patient developed black pigmentation on the tongue accompanied by dysgeusia, negatively affecting the quality of life. Discontinuing minocycline resulted in a gradual improvement of these symptoms. This report underscores the importance of clinicians being vigilant to the risk of black pigmentation on the tongue in patients receiving long-term tetracycline antibiotics for the treatment of EGFR inhibitor-induced skin lesions.

## Introduction

Skin lesions caused by epidermal growth factor receptor (EGFR) inhibitors, such as panitumumab and cetuximab, are adverse effects that may result in chemotherapy dose reduction or discontinuation [1]. Combining panitumumab with multiple chemotherapy regimens has demonstrated efficacy in substantially improving overall survival; it is a standard first-line treatment for wild-type Kristen rat sarcoma virus (KRAS) and left-sided metastatic colorectal cancer (mCRC) [2]. Patients exhibiting skin lesions because of EGFR inhibitors often require long-term management involving oral antibiotics and topical adrenocorticosteroids [3]. Despite adequate skin care, antibacterial and anti-inflammatory effects of tetracycline antibiotics are recommended for EGFR-induced acne dermatitis [4]. However, antibiotic use for preventing or treating infection may result in pigmentation on the tongue and especially turn the color black [5]. Herein, we present a 68-year-old man with mCRC who experienced black pigmentation on the tongue owing to long-term minocycline use for skin lesions induced by chemotherapy involving panitumumab combined with irinotecan, folic acid, and 5-fluorouracil (FOLFIRI).

## Case presentation

In early April 2021, a 68-year-old, nonsmoking Japanese man with a history of hepatitis B infection, hypertension, and prostatic hyperplasia presented with left lower abnormal pain and had a checkup at a local clinic in Yokohama, Japan. His family history included pancreatic cancer in his mother. Colonoscopy revealed a type 2 semicircular tumor in a 12-cm area corresponding to the anal verge. Subsequently, the patient was referred to the Digestive Disease Center Department of Showa University Northern Yokohama Hospital, Yokohama, Japan. In May 2021, the patient was diagnosed with cStage IIIb sigmoid colon cancer (cT3, N1, M0) based on computed tomography (CT) scans of the chest, abdomen, and pelvis.

After one month, laparoscopic high anterior resection was performed. Following a pathological examination indicating pStage III (pT3, N3, M0) classification, postoperative adjuvant chemotherapy with capecitabine (Cape) and oxaliplatin (OX) (Cape 3,600 mg/body: days 1-14 + OX 130 mg/m^2^: day 1: every three weeks) commenced one month after surgery. Within six months after completing eight courses of Cape + OX chemotherapy, the patient was diagnosed with metastases to the lung, liver, and adrenal gland through positron emission tomography CT. The KRAS/B-rapidly accelerated fibrosarcoma (BRAF)/microsatellite instability (MSI) genetic mutation test showed that the patient had a wild-type KRAS/BRAF and MSI-low status.

Panitumumab combined with the FOLFIRI regimen (panitumumab 6 mg/kg, irinotecan 150 mg/m^2^, folinic acid 200 mg/m^2^, 5-fluorouracil 400 mg/m^2^, intravenous infusion on day 1, 5-fluorouracil 2400 mg/m^2^, continuous intravenous infusion on day 1 for 48 hours, 14 days per cycle) was selected for systemic chemotherapy. This selection was based on considerations of potential efficacy, confirmed safety, prior treatment, gene mutation, and tumor site. These chemotherapies, administered at outpatient visits, commenced 1.5 years after diagnosis.

The patient did not experience any specific adverse events before chemotherapy. After four courses of panitumumab + FOLFIRI regimen, grade 3 skin lesions appeared on the lower legs and back, accompanied by paronychia. Following dermatologic consultation, the patient was first prescribed minocycline 100 mg and steroid ointments (betamethasone valerate and gentamicin sulfate ointment 0.12% and clobetasol propionate 0.05%) to improve the skin lesions. Subsequently, three months into the skin lesion treatments, the minocycline dose was increased to 200 mg owing to the spread of the lesions on the legs, manifesting as dark purple, deep red spots approximately the size of soybean and worsening of paronychia, accompanied by nail cracks, causing a change in color to pale dark red or dark reddish brown. One month after the dose increase, with improvements observed in the skin lesions, the minocycline dose was decreased to 100 mg.

**Figure 1:**
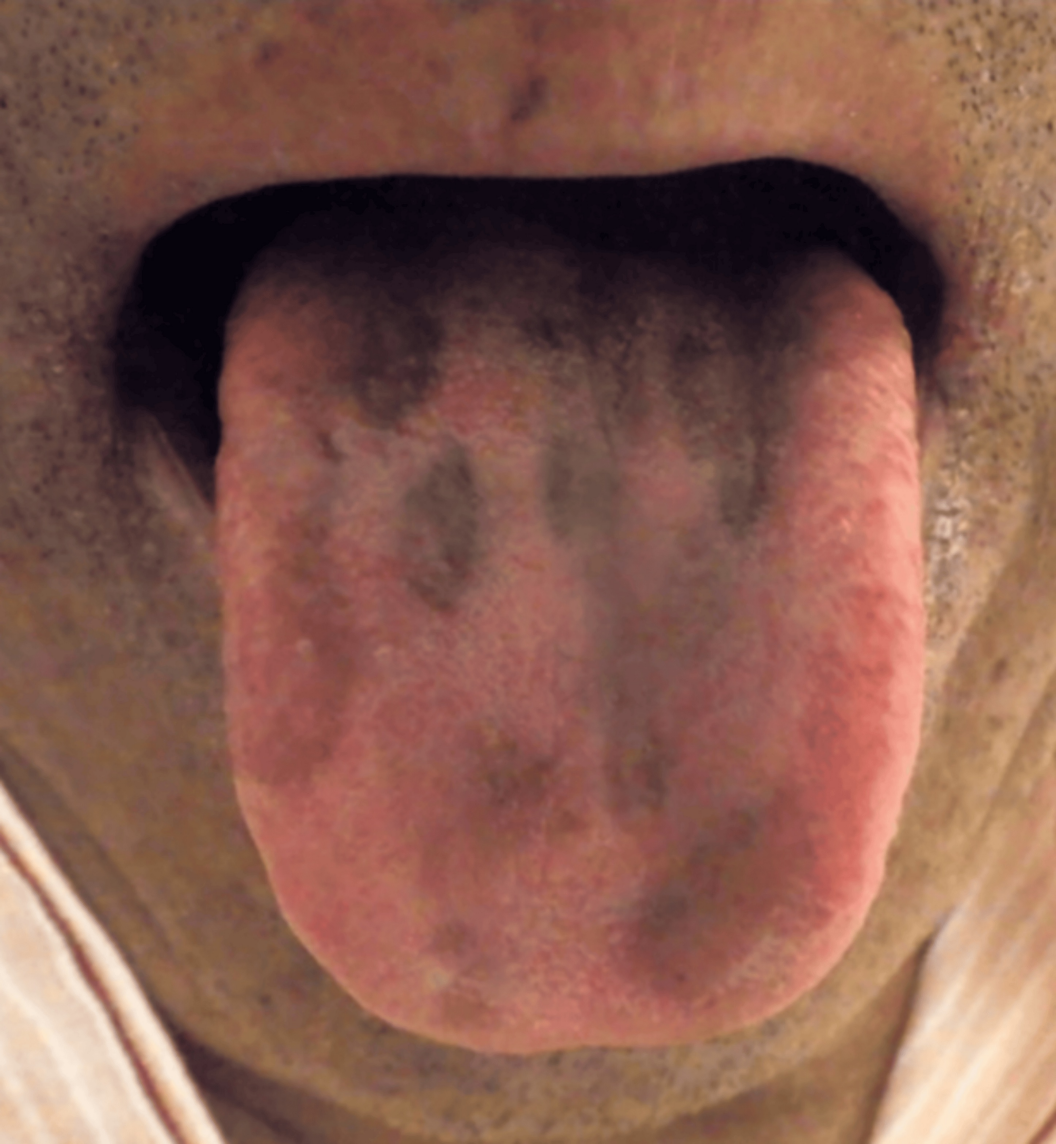
Six months after minocycline administration showing black coloration of the tongue of the patient

**Figure 2:**
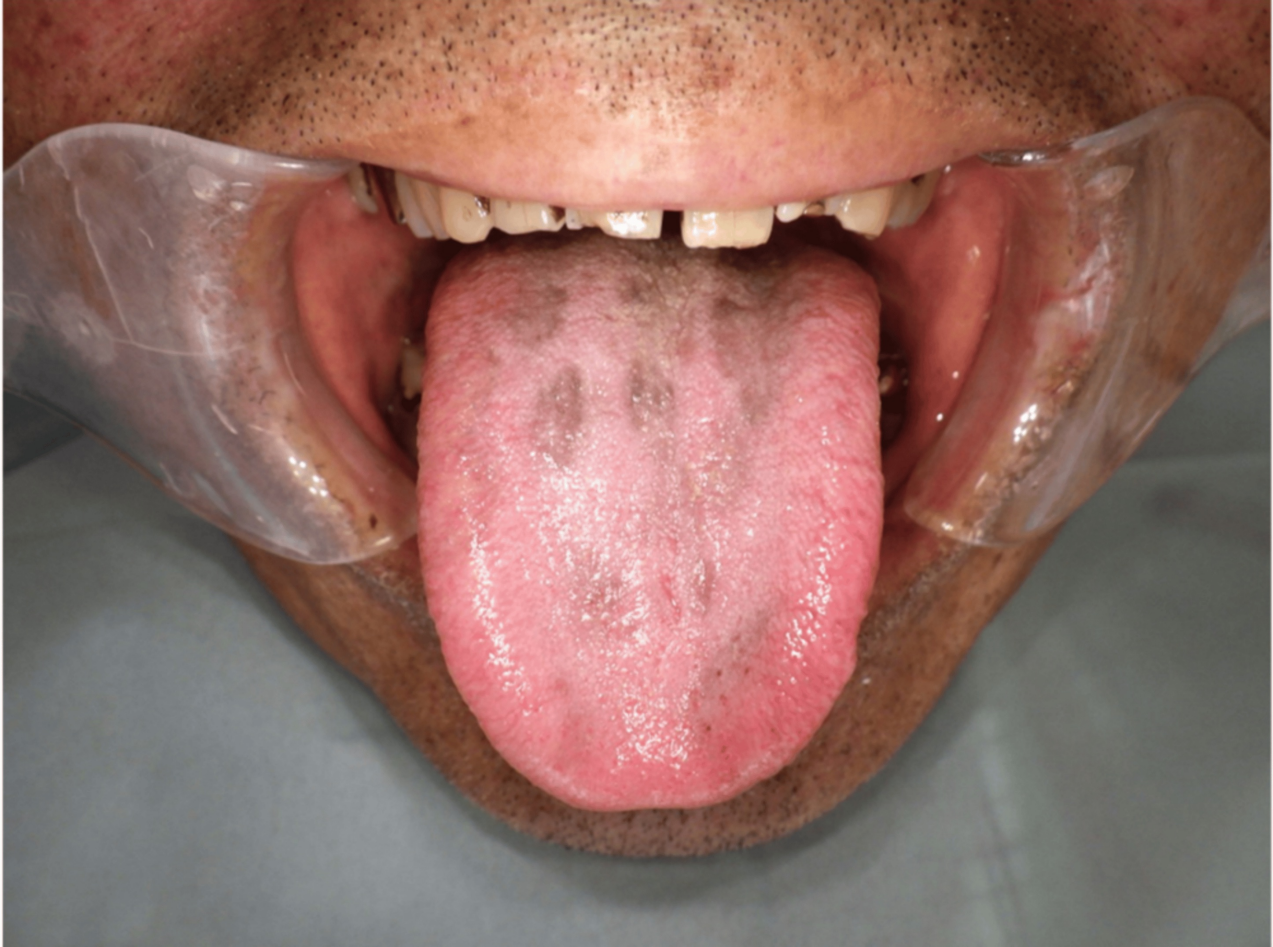
Two weeks after discontinuing minocycline when diagnosed with black pigmentation on the tongue by a dentist

**Figure 3:**
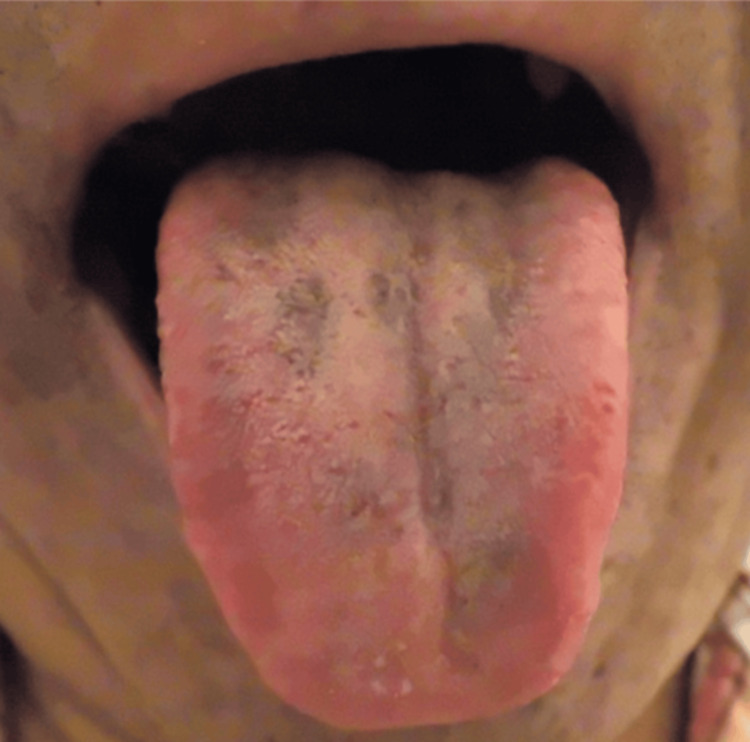
A follow-up conducted six weeks after discontinuing minocycline, demonstrating a normalization of the color in the dorsal area tongue

However, in July 2023, after six months of minocycline treatment, the patient complained of worsening dysgeusia to grade 2. Intraoral examination revealed a darkened and black appearance of the middle to the posterior tongue (Figure 1). We suspected a minocycline-related adverse effect based on a score of 7 on the Naranjo scale (Table 1) [6] and additionally applied the World Health Organization The Uppsala Monitoring Centre (WHO-UMC) causality assessment scale as it is widely recognized by international regulatory authorities and pharmacovigilance systems [7]. In this case, minocycline can be categorized as “possible,” while chemotherapy medicines can be categorized as “unlikely” by the WHO-UMC causality assessment because of the temporal relationship. Therefore, minocycline was discontinued, but other medications, including chemotherapy, were maintained. The patient was diagnosed with black pigmentation on the tongue following a dental consultation. However, the color of the dorsal tongue and dysgeusia gradually normalized within two weeks after discontinuing minocycline (Figures 2, 3).

**Table 1:** Naranjo adverse drug reaction probability scale The adverse drug reaction is assigned to a probability category from the total score as follows: definite if the overall score is 9 or greater, probable for a score of 5-8, possible for 1-4, and doubtful if the score is 0

Questions	Yes	No	Do not know	Score
Are there previous conclusive reports on this reaction?	1	0	0	1
Did the adverse event occur after the suspected drug was administered?	2	-1	0	2
Did the adverse reaction improve when the drug was discontinued or a specific antagonist was administered?	1	0	0	1
Did the adverse reaction reappear when the drug was readministered?	2	-1	0	0
Are there alternative causes (other than the drug) that could have on their own caused the reaction?	-1	2	0	2
Did the reaction reappear when a placebo was given?	-1	1	0	0
Was the blood test detected in the blood (or the fluid) in concentrations known to be toxic?	1	0	0	0
Was the reaction more severe when the dose was increased or less severe when the dose was decreased?	1	0	0	0
Did the patient have a similar reaction to the same or similar drugs in any previous exposure?	1	0	0	0
Was the adverse event confirmed by any objective evidence?	1	0	0	1
Total	-	-	-	7

Presently, chemotherapy is continued, whereas panitumumab administration is repeatedly started and stopped according to the severity of acneiform efflorescence and paronychia. This case report conforms to the CAse REports guidelines, and the authors have obtained informed consent from the patient for the publication of patient information and images.

## Discussion

Our case indicates a strong relationship between black pigmentation on the tongue and minocycline in a colorectal cancer patient. Minocycline-induced black pigmentation on the tongue is thought to occur through multiple mechanisms. Based on previous studies [8,9], the involvement of minocycline, particularly with long-term use, in pigment formation suggests that alteration of the oral microbiome due to antibiotics, including minocycline, melanin deposition resulting from the effects of minocycline-metabolite complexes, and the interaction of minocycline with metal ions collectively explain the minocycline-induced pigment deposition in the skin and oral mucosa observed in patients. In addition, the symptoms include dysgeusia, halitosis, and oral discomfort. Therefore, long-term minocycline use was considered a potential cause of black pigmentation on the tongue and dysgeusia in our case.

Through a literature review, we identified 11 cases of antibiotic-induced black pigmentation on the tongue in adults between 2019 and 2023. Patients aged 30-80 years developed black pigmentation on the tongue seven days to 11 weeks after receiving the causative drug, with symptoms improving within one week to three months after drug withdrawal. Minocycline-induced black pigmentation on the tongue was specifically described in two of these reports [10,11]. In all cases, discontinuing the causative antibiotics improved black pigmentation on the tongue (Table 2) [10-17]. While black pigmentation on the tongue reportedly occurs following antibiotic administration for infection, the antibiotic administration period in the literature on patients with cancer, which is similar to our case, has not been clearly described [10]. Minocycline, typically administered for six to eight weeks to prevent EGFR inhibitor-induced skin lesions [18], may be administered long term based on symptoms. In our case, minocycline was administered for six months, which is longer than the duration reported previously. Our analysis suggests that the long-term use of minocycline for skin lesions, rather than infections, may cause black pigmentation on the tongue. Consistent with previous cases, black pigmentation on the tongue improved upon causative drug discontinuation. Therefore, in the event of black pigmentation on the tongue, discontinuing tetracycline antibiotics may be the viable treatment option. However, monitoring patients for any exacerbation of EGFR inhibitor-induced skin lesions is essential.

**Table 2:** Literature review of antibiotic-induced black pigmentation on the tongue/black hairy tongue in adults between 2019 and 2023

Case no.	Study	Year	Patient characteristics	Causative drug	Period from initiating causative drug to the onset of black pigmentation on the tongue	Duration of improved black pigmentation on the tongue episode	Treatment for black pigmentation on the tongue	Treatment results for black pigmentation on the tongue
1	Takata and Hirai [10]	2023	A 60-year-old woman with hepatitis B and rectal cancer	Minocycline	Not listed clearly	Six weeks	Discontinuation of minocycline	Improved
2	Sakaguchi and Watari [11]	2020	A 73-year-old woman with Japanese spotted fever or tsutsugamushi infection	Minocycline	Administered seven days and discontinued five days	Not listed	Self-limiting	Improved
3	Okumura and Kawashima [12]	2023	An 80-year-old woman with diabetes mellitus and renal abscess	Ampicillin-sulbactam	28 days	Three months	No change in antibiotics, and good oral hygiene	Improved
4	Shangguan et al. [13]	2022	A 74-year-old man with lung adenocarcinoma and tuberculosis	Linezolid	12 days	17 days	Discontinuation of linezolid	Improved
5			A 67-year-old man with tuberculosis	Linezolid	13 days	10 days	Completing administration for two months	Improved
6			A 37-year-old man with tuberculosis	Linezolid	28 days	15 days	Discontinuation of linezolid	Improved
7	Niiyama and Hase [14]	2021	An 82-year-old woman with brain infection	Metronidazole	28 days	Two weeks	Discontinuation of metronidazole	Improved
8	Lee et al. [15]	2021	A 60-year-old woman with tuberculosis	Linezolid	11 weeks	Three weeks	Discontinuation of linezolid	Improved
9	Ren et al. [16]	2020	A 17-year-old girl with a central neurocytoma	Piperacillin-tazobactam	12 days	Eight days	Discontinuation of piperacillin-tazobactam	Improved
10			A 65-year-old man with multidrug-resistant *Pseudomonas aeruginosa* infection	Piperacillin-tazobactam	15 days	13 days	Discontinuation of piperacillin-tazobactam and levofloxacin	Improved
11	Zhao et al. [17]	2019	A 39-year-old man with diabetic foot infection	Imipenem/cilastatin	Two weeks	One week	Discontinuation of imipenem/cilastatin	Improved

A few reports suspected chemotherapy-induced black pigmentation on the tongue [19]. However, black pigmentation on the tongue occurred immediately within two months after the initiation of chemotherapy in solid tumor patients, and it was fully resolved upon the withdrawal of chemotherapy medications, while in this case, chemotherapy was continued for approximately seven months. In addition, black pigmentation on the tongue improved with the discontinuation of minocycline despite the continuation of other medications, including chemotherapy. Therefore, the temporal relationship between minocycline discontinuation and symptom improvement strongly suggests that minocycline, rather than chemotherapy medications, was the more likely cause of black pigmentation on the tongue.

Taste disorders can arise from various factors, including chemotherapy-induced taste neuropathy, zinc deficiency, mouth ulcers, and intraoral dryness. However, the pathology of taste disorders remains unclear, and effective treatments are not well-established, leading to poor nutritional status owing to anorexia, which affects quality of life [20]. In our case, the decrease in food intake due to dysgeusia was initially suspected to be a chemotherapy-induced adverse event because the patient was administered OX, irinotecan, and 5-fluorouracil in multiple instances. However, it was later considered that dysgeusia might be associated with black pigmentation on the tongue when the patient complained of black coloration of his tongue. Therefore, in patients with cancer presenting with dysgeusia, clinicians should comprehensively evaluate chemotherapy-induced adverse events. Furthermore, clinicians should be vigilant about the risk of black pigmentation on the tongue in patients who are administered tetracycline antibiotics in the long term to prevent EGFR inhibitor-induced skin lesions.

## Conclusions

In conclusion, our case indicates a strong relationship between black pigmentation on the tongue and the long-term use of minocycline for the treatment of EGFR inhibitor-induced skin lesions in a colorectal cancer patient. Our findings suggest that discontinuation of minocycline can effectively resolve both black pigmentation on the tongue and the associated dysgeusia. This underscores the need for clinicians to be vigilant when prescribing tetracycline antibiotics, especially long-term antibiotics, for the treatment of EGFR inhibitor-induced skin lesions. Early recognition of black pigmentation on the tongue and prompt discontinuation of the causative antibiotic may prevent further complications and improve patient quality of life. In addition, a comprehensive evaluation of chemotherapy-induced and medication-related adverse events is crucial for optimizing care in cancer patients.
